# Skeletal muscle analysis of cancer patients reveals a potential role for carnosine in muscle wasting

**DOI:** 10.1002/jcsm.13258

**Published:** 2023-05-18

**Authors:** Dheeraj Kumar Posa, Janice Miller, David Hoetker, Michael I. Ramage, Hong Gao, Jingjing Zhao, Benjamin Doelling, Aruni Bhatnagar, Stephen J. Wigmore, Richard J.E. Skipworth, Shahid P. Baba

**Affiliations:** ^1^ Center for Cardiometabolic Science Louisville Kentucky USA; ^2^ Christina Lee Brown Envirome Institute Louisville Kentucky USA; ^3^ Department of Clinical Surgery University of Edinburgh Edinburgh UK

**Keywords:** Anserine, Beta‐alanine, Histidyl dipeptides, Lipid peroxidation products, Ubiquitin

## Abstract

**Background:**

Muscle wasting during cancer cachexia is mediated by protein degradation *via* autophagy and ubiquitin‐linked proteolysis. These processes are sensitive to changes in intracellular pH ([pH]_i_) and reactive oxygen species, which in skeletal muscle are partly regulated by histidyl dipeptides, such as carnosine. These dipeptides, synthesized by the enzyme carnosine synthase (CARNS), remove lipid peroxidation‐derived aldehydes, and buffer [pH]_i_. Nevertheless, their role in muscle wasting has not been studied.

**Methods:**

Histidyl dipeptides in the rectus abdominis (RA) muscle and red blood cells (RBCs) of male and female controls (*n* = 37), weight stable (WS: *n* = 35), and weight losing (WL; *n* = 30) upper gastrointestinal cancer (UGIC) patients, were profiled by LC–MS/MS. Expression of enzymes and amino acid transporters, involved in carnosine homeostasis, was measured by Western blotting and RT‐PCR. Skeletal muscle myotubes were treated with Lewis lung carcinoma conditioned medium (LLC CM), and β‐alanine to study the effects of enhancing carnosine production on muscle wasting.

**Results:**

Carnosine was the predominant dipeptide present in the RA muscle. In controls, carnosine levels were higher in men (7.87 ± 1.98 nmol/mg tissue) compared with women (4.73 ± 1.26 nmol/mg tissue; *P* = 0.002). In men, carnosine was significantly reduced in both the WS (5.92 ± 2.04 nmol/mg tissue, *P* = 0.009) and WL (6.15 ± 1.90 nmol/mg tissue; *P* = 0.030) UGIC patients, compared with controls. In women, carnosine was decreased in the WL UGIC (3.42 ± 1.33 nmol/mg tissue; *P* = 0.050), compared with WS UGIC patients (4.58 ± 1.57 nmol/mg tissue), and controls (*P* = 0.025). Carnosine was significantly reduced in the combined WL UGIC patients (5.12 ± 2.15 nmol/mg tissue) compared with controls (6.21 ± 2.24 nmol/mg tissue; *P* = 0.045). Carnosine was also significantly reduced in the RBCs of WL UGIC patients (0.32 ± 0.24 pmol/mg protein), compared with controls (0.49 ± 0.31 pmol/mg protein, *P* = 0.037) and WS UGIC patients (0.51 ± 0.40 pmol/mg protein, *P* = 0.042). Depletion of carnosine diminished the aldehyde‐removing ability in the muscle of WL UGIC patients. Carnosine levels were positively associated with decreases in skeletal muscle index in the WL UGIC patients. CARNS expression was decreased in the muscle of WL UGIC patients and myotubes treated with LLC‐CM. Treatment with β‐alanine, a carnosine precursor, enhanced endogenous carnosine production and decreased ubiquitin‐linked protein degradation in LLC‐CM treated myotubes.

**Conclusions:**

Depletion of carnosine could contribute to muscle wasting in cancer patients by lowering the aldehyde quenching abilities. Synthesis of carnosine by CARNS in myotubes is particularly affected by tumour derived factors and could contribute to carnosine depletion in WL UGIC patients. Increasing carnosine in skeletal muscle may be an effective therapeutic intervention to prevent muscle wasting in cancer patients.

## Introduction

Cancer‐induced cachexia is an insidious syndrome characterized by progressive loss of skeletal muscle and fat. It is associated with diminished tolerance to chemotherapy and anticancer drugs, and it has dramatic effects on quality and length of life.^1^, ^2^, ^3^ Currently, there are no effective nutritional or pharmacological approaches that fully prevent or reverse body weight loss in cancer patients. Moreover, no easily identifiable biomarkers of cancer cachexia are available that can faithfully monitor or reflect cachectic muscle pathophysiology.

The full reasons why cancer results in muscle atrophy remain unknown. A rapid loss of body weight in cancer patients is usually associated with a shift in muscle fuel utilization,^4^ and hypermetabolism induced by tumour derived factors, but the relative contribution of these factors remains unclear. However, once begun, muscle atrophy is accompanied by protein hypercatabolism, a decrease in protein anabolism, and an activation of protein degradation pathways, such as the ubiquitin proteasome pathway and autophagy. Several studies have shown that these protein degradation pathways are activated by oxidative stress,^5^, ^6^, ^7^ and a decrease in intracellular pH ([pH]_i_).^8^, ^9^ Excessive generation of reactive oxygen species (ROS) alters calcium homeostasis and activates cysteine protease calpains,^10^ increases enzymatic activity and expression of ubiquitin/proteasome,^5^ and stimulates autophagy.^11^, ^12^, ^13^ Similarly, acidosis increases the expression of mRNAs that encode ubiquitin proteasome subunits, and enhances the rate of proteolysis in muscle.^8^

Oxidative stress in skeletal muscles in countered by a range of antioxidant defences, including ROS scavenging enzymes, such as superoxide dismutase, catalase and peroxiredoxin,^14^ whereas pH homeostasis is maintained by different transporters, such as lactate/H^+^ and Na^+^/H^+^ transporters.^15^ In addition, skeletal muscles contain high (5–10 mM) levels of histidyl dipeptides, such as carnosine (β‐alanine‐histidine), and anserine (β‐alanine‐N^π^‐histidine).^16^ As the pK_a_ of amino group of histidine in these dipeptides is near [pH] 6.0, these peptides can efficiently buffer intracellular protons generated during ischemia or prolonged bouts of exercise.^17^, ^18^ Moreover, because the amino group of these peptides readily forms Schiff bases or Michael adducts with strong electrophiles, these dipeptides can also scavenge reactive lipid peroxidation products, such as acrolein, the downstream products of oxidative stress.^16^ Given the high sensitivity of protein degradation pathways to both oxidative stress and intracellular buffering, it is likely that the levels of histidyl dipeptides are important regulators of muscle wasting such as during cancer‐induced cachexia, but this role has never been studied. Accordingly, the aim of this study was to examine how tumour stress affects histidyl dipeptides and how these peptides affect protein degradation pathways during tumour‐induced muscle wasting. We also assessed whether carnosine levels in red blood cells (RBCs) could be an informative biomarker of muscle loss.

## Methods

### Study participant

Patients with histologically confirmed upper gastrointestinal cancer (UGIC) suitable for surgical resection were recruited from the regional multidisciplinary team meeting (*n* = 65), including patients who were weight stable (WS, *n* = 35), and weight losing (WL, *n* = 30) (consistent with a diagnosis of cancer cachexia based on the 2011 consensus definition^1^). Controls recruited (*n* = 37) were patients undergoing elective abdominal procedures (repair of aortic aneurysm or donor nephrectomy). All patients were able to provide written consent and the study was approved by the ethics committee (UK).

### Body composition analysis

Skeletal muscle index (SMI) was calculated either from routine staging CT scans performed prior to any surgical intervention, or from the post chemotherapy re‐staging scan if the patients underwent neoadjuvant chemotherapy. Digital CT images obtained with a spiral CT scanner were analysed using Slice‐O‐matic and ABACS software. SMI was derived from measurements of muscle cross sectional area normalized to body stature (cm^2^/m^2^) at the level of 3^rd^ lumbar vertebra (L3). Sex‐specific SMI cut‐offs for low SMI were obtained as described in the published literature^19^ (see supporting information).

### Blood measures

Plasma C‐reactive protein (CRP), was measured using the highly sensitive enzyme linked immunosorbent assay (ELISA, Ely, UK) following the manufacturers protocol as published previously.^20^ A CRP ≥ 5 mg/L was considered to be reflective of systemic inflammation.

### Muscle biopsy and red blood cells collection

Muscle biopsies were collected under general anaesthesia at the start of surgical procedure following the opening of abdomen (see supporting information). Venous blood samples, ~5 mL, drawn into heparinized tubes were taken at induction of anaesthesia. Blood samples were centrifuged at 1000*× g* for 15 min at room temperature to separate plasma from RBCs and WBCs.

### Histidyl dipeptide profiling of rectus abdominis muscle and red blood cells

Muscle biopsies and RBCs collected from the subjects were analysed for different histidyl dipeptides and aldehyde conjugates by performing UPLC‐ESI‐MS/MS (see supporting information).

### Western blotting analysis

RA muscle samples (*n* = 10 in each group) were homogenized in radioimmunoprecipitation assay buffer, centrifuged for 25 min at 13 000*× g*, and the supernatants were separated by SDS‐PAGE, and immunoblots were developed with different antibodies (see supporting information).

### Quantitative polymerase chain reaction

Changes in the mRNA levels were measured by isolating total RNA from the RA muscle using RNeasy Fibrous Tissue Mini kit (Qiagen) (see supporting information).

### Treatment of the murine myotubes (differentiated C2C12 cells) with Lewis lung carcinoma conditioned medium (LLC CM)

C2C12 cells were maintained in Dulbecco's modified Eagle's medium (DMEM), 10% fetal bovine serum (FBS) and 0.1% penicillin/streptomycin.

### Statistical analysis

Descriptive statistics were determined for all histidyl dipeptides, carnosine aldehyde conjugates, enzymes and amino acid transporters, and clinical parameters [body mass index (BMI), skeletal mass index (SMI), C‐reactive protein (CRP) and age]. GraphPad analysis software and SAS (version 9.4, SAS Institute, Inc., Cary, North Carolina) were used for statistical analysis. Proportions are expressed in percentages. Chi‐square test or Fisher's exact test as appropriate was used to compare differences in proportions for sex, cancer type, tumour stage and nodal classification. Differences in histidyl dipeptides, carnosine aldehydes conjugates and clinical parameters in the men and women, and protein and gene expression, in the three groups; controls, WS and WL UGIC patients were estimated by a two‐way ANOVA followed by post hoc Tukey's analysis. For the combined analysis of men and women, a multiple variable linear regression model was built using PROC GLM and differences in histidyl dipeptides, carnosine aldehydes conjugates were performed using one‐way ANOVA analysis. Both sex and patient group were included as independent variables, whereas carnosine and carnosine aldehyde conjugates as dependent variables. Spearman correlations were performed across 4 dipeptides and 2 carnosine aldehyde conjugates and 4 clinical characteristics including BMI, age, CRP and SMI in the controls (*n* = 37), WL UGIC (*n* = 35) and the WS UGIC patients (*n* = 30). Heat map for the Spearman correlation coefficients was generated in R (Version 4.0.5). Association between the RBCs and muscle carnosine in the WL UGIC patients was examined using linear regression. Statistical significance was accepted at *P* < 0.05. Data are expressed as mean ± standard deviation (SD).

## Results

### Clinical characterization of upper gastrointestinal cancer patients with and without cachexia

Patient characteristics are depicted in Table 1. One hundred and two patients were recruited and categorized into three groups: controls, WS upper gastrointestinal cancer (UGIC) patients and weight losing (WL) UGIC patients. In the control subjects, there are 46% men and 54% women and in the cancer patient cohort, there were 63% men that was higher compared with 37% women. Compared with the controls (53 ± 12 years), WS UGIC (63 ± 9 years) and WL UGIC patients were older (71 ± 8 years; *P* = 0.009). CRP was significantly higher in both the WS (18.42 ± 16 mg/mL; *P* = 0.002), and WL UGIC patients (16.52 ± 14 mg/mL; *P* = 0.007) compared with the controls (3.72 ± 4 mg/mL). In men, WS UGIC patients showed a mean body weight loss of 0.4 ± 1% (*P* = 0.082), whereas WL UGIC patients had a mean body weight loss of 8.33 ± 4% (*P* = 0.001) over the past 6 months. In women, WS UGIC patients showed a mean body weight loss of 0.93 ± 1% (*P* = 0.005 vs. controls), whereas WL UGIC patients had a mean body weight loss of 5.93 ± 4.85% (*P* = 0.001 vs. controls). Moreover, SMI measured by CT was lower in the WL UGIC patients (44 ± 7 cm^2^/m^2^) compared with the controls (47 ± 7 cm^2^/m^2^; *P* = 0.009), but did not reach statistical significance compared with WS UGIC patients (49 ± 10 cm^2^/m^2^; *P* = 0.161).

**Table 1:** Demographic characteristic of the patient population.

Clinical characteristics	Control (*n* = 37)	Weight stable (*n* = 35)	Weight losing (*n* = 30)	*P*‐value
Age (years)	53 ± 12	63 ± 9	71 ± 8	<0.05* ^,^ ^#^
Sex				
Male (%)	17 (46)	22 (63)	19 (63)	0.243
Female (%)	20 (54)	13 (37)	11 (38)	
Cancer type				0.140
Oesophageal adenoid cystic carcinoma		25 (72)	16 (53)	
Gastric adenoid cystic carcinoma		7 (20)	9 (30)	
Pancreatic		2 (5)	3 (10)	
Gist lymphoma		1 (3)		
Cholangiocarcinoma			2 (7)	
Tumour stage				0.798
T1a		4 (12)	1 (4)	
T1b		8 (24)	10 (33)	
T2		4 (12)	7 (23)	
T3		16 (44)	9 (30)	
T1S		2 (6)		
T4a		1 (3)	2 (6)	
T4b			1 (4)	
Nodal classification				0.280
N0		18 (52)	9 (30)	
N1		8 (22)	15 (60)	
N2		4 (11)	3 (10)	
N3		5 (15)	3 (10)	
Neoadjuvant chemotherapy		6	4	
BMI (kg/m^2^)	25.6 ± 3.6	28.3 ± 6.6	26.1 ± 4.9	NS
% Weight loss		0.7 ± 1	7.64 ± 5	<0.04* ^,^ ^#^
SMI (cm^2^/m^2^)	47 ± 7	49 ± 10	44 ± 7	<0.009*
CRP (mg/L)	3.72 ± 4	18.42 ± 16	16.52 ± 14	<0.05* ^,^ ^#^

Body mass index (BMI), skeletal muscle index (SMI), and C‐reactive protein (CRP) for the controls, weight stable (WS) and weight losing (WL) upper gastrointestinal cancer patients are presented as mean ± SD. Cancer type, tumour stage, and nodal classification are presented as proportions. NS is not significant.

* *P* < 0.05 versus controls.

^#^ *P* < 0.05 versus cancer WS patients.

### Effect of upper gastrointestinal cancer on histidyl dipeptides levels in the male and female skeletal muscle

**Figure 1:**
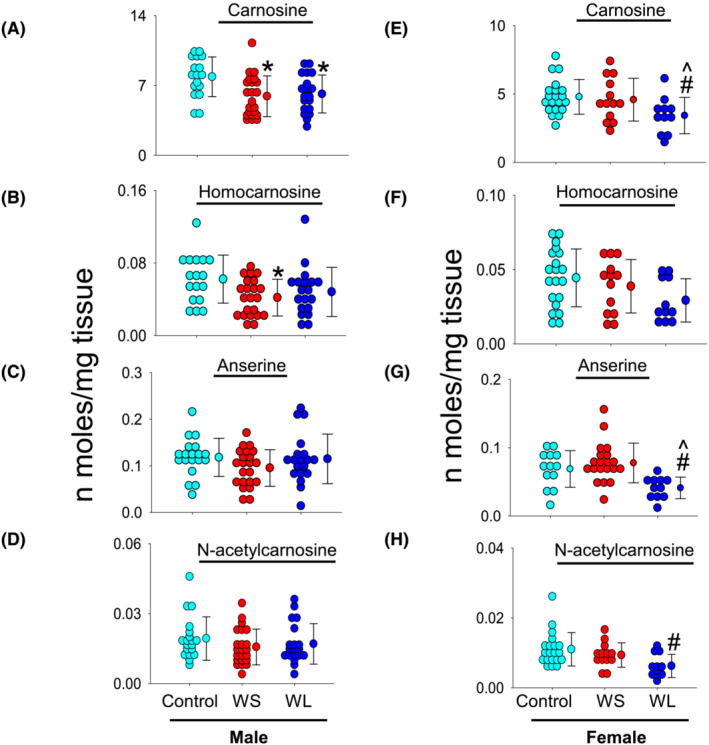
Histidyl dipeptide levels in the rectus abdominus muscle. Tissues collected from the male (*n* = 17) and female controls (*n* = 20), weight stable (WS) upper gastrointestinal cancer (UGIC) male (*n* = 22), and female (*n* = 13) patients, and weight losing (WL) UGIC male (*n* = 19) and female (*n* = 11) patients. Tissues were analysed by LC–MS/MS for different histidyl dipeptides. Levels of (A, E) carnosine, (B, F) homocarnosine, (C, G) anserine, and (D, H) N‐acetyl carnosine in male and female subjects. Data are shown as mean ± SD, *P = 0.009 and 0.030 for carnosine in the WS and WL UGIC patients respectively versus male controls, ^δ^ *P* = 0.033 in WS UGIC subjects versus control. In women, for carnosine ^*P* = 0.050 in the WL versus WS UGIC female subjects and ^#^ *P* = 0.025 in the WL UGIC subject's versus female controls. For anserine, ^*P* = 0.008 in the WL versus WS UGIC female subjects and ^#^ *P* = 0.009 in the WL UGIC subject's versus female controls. For N‐acetyl carnosine, ^#^ *P* = 0.008 in the WL UGIC subject's versus female controls.

We performed a comprehensive histidyl dipeptide mapping of the rectus abdominus (RA) muscle in the male and female subjects. Among the different histidyl dipeptides, carnosine was the predominant dipeptide. Its levels were approximately 1–2‐fold higher in male (7.87 ± 1.98 nmol/mg tissue) compared with female controls (4.79 ± 1.26 nmol/mg tissue; *P* = 0.002, Figure 1A,E). Next, we compared the histidyl dipeptide levels between the male controls, WS and WL UGIC patients. Levels of carnosine were lower in the WS (5.92 ± 2.04 nmol/mg tissue; *P* = 0.009) and WL UGIC patients (6.15 ± 1.90 nmol/mg tissue; *P* = 0.030) compared with the controls (Figure 1A). Homocarnosine levels in the WS UGIC patients (0.041 ± 0.020 nmol/mg tissue) were significantly lower compared with controls (0.062 ± 0.026 nmol/mg tissue; *P* = 0.033), whereas in the WL UGIC patients homocarnosine was lower (0.048 ± 0.027 nmol/mg tissue, Figure 1B) compared with the controls, but unable to reach statistical significance. Levels of anserine and N‐acetyl carnosine remained unchanged between the different groups (Figure 1C,D). In women, carnosine levels in the WL UGIC patients were decreased (3.42 ± 1.33 nmol/mg tissue) compared with the WS UGIC patients (4.58 ± 1.57 nmol/mg tissue; *P* = 0.050), and the controls (carnosine: 4.79 ± 1.26 nmol/mg tissue; *P* = 0.025, Figure 1E). Similarly, in the WL UGIC patients, levels of anserine (0.041 ± 0.016 nmol/mg tissue) were significantly lower than in WS UGIC patients (0.069 ± 0.027 nmol/mg tissue; *P* = 0.008) and controls (0.078 ± 0.029 nmol/mg tissue; *P* = 0.009, Figure 1G). N‐acetyl carnosine was significantly lower in WL UGIC patients (0.006 ± 0.003 nmol/mg tissue) than in controls (0.011 ± 0.004 nmol/mg tissue; *P* = 0.008), but remained unaffected in the WS UGIC patients (0.009 ± 0.003 nmol/mg tissue; Figure 1H). No change in homocarnosine levels was observed between the three groups (Figure 1F).

### Effect of upper gastrointestinal cancer on the histidyl dipeptides in skeletal muscle

**Figure 2:**
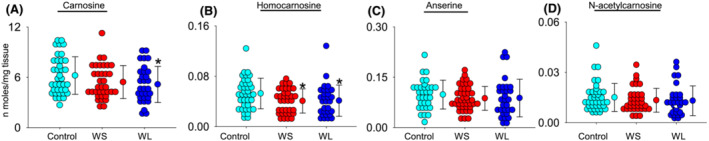
Histidyl dipeptide levels combined in the male and female subjects. Levels of (A) carnosine, (B) homocarnosine, (C) anserine, and (D) N‐acetylcarnosine collected from the male and female controls (*n* = 37), weight stable (WS) upper gastrointestinal cancer (UGIC; *n* = 35) and weight losing (WL) UGIC (*n* = 30) patients. Data are presented as mean ± SD, *P = 0.045 for carnosine in the WL UGIC patients versus the controls, ^#^ *P* = 0.029 and 0.045 for homocarnosine in the WS and WL UGIC patients versus the controls.

Next, to examine which dipeptide in both sexes are predominantly affected in cancer patients, we combined data from the male and female subjects and compared all the histidyl dipeptides. Notably, in comparison with other histidyl dipeptides, carnosine was the only dipeptide that was significantly decreased (5.12 ± 2.15 nmol/mg tissue) in the RA muscle of WL UGIC patients compared with controls (6.21 ± 2.24 nmol/mg tissue; *P* = 0.045), whereas no difference was observed in the WS UGIC patients (5.42 ± 1.96 nmol/mg tissue, Figure 2A). Homocarnosine was significantly decreased in WS UGIC patients (0.040 ± 0.019 nmol/mg tissue; *P* = 0.029) and WL UGIC patients (0.041 ± 0.024 nmol/mg tissue; *P* = 0.045) compared with controls (0.052 ± 0.02 nmol/mg tissue, Figure 2B). Levels of the other dipeptides anserine and N‐acetylcarnosine remained unchanged (Figure 2C,D).

### Carnosine measurements in red blood cells and correlation with muscle carnosine

**Figure 3:**
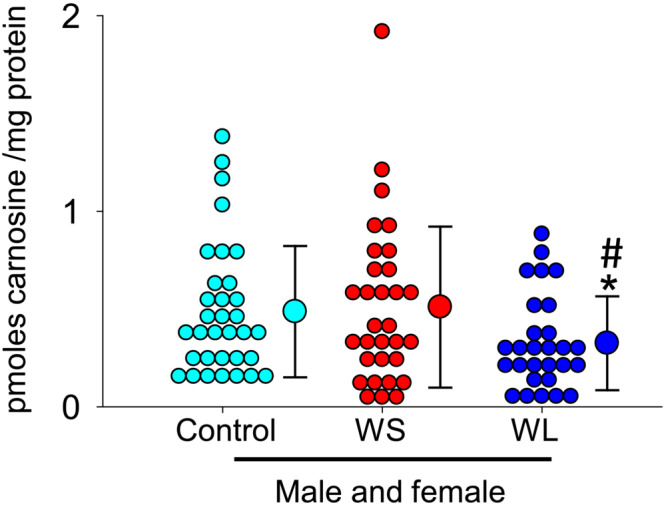
Carnosine measurements in the red blood cells (RBCs). Carnosine in the RBCs collected from male and female controls (*n* = 37), weight stable (WS) (*n* = 35) and weight losing (WL) upper gastrointestinal cancer (*n* = 30) patients. Data are shown as mean ± SD, *P = 0.037 and 0.042 in the WL UGIC patients versus the controls and WS UGIC patients, respectively.

We next examined whether carnosine in RBCs could be a diagnostic muscle wasting biomarker. No differences in carnosine levels were observed between the three groups, when male and female subjects were analysed separately (data not shown). However, when all the subjects were combined, carnosine levels in the RBCs of WL UGIC patients (men and women) were lower (0.32 ± 0.24 pmol/mg protein) compared with controls (0.49 ± 0.31 pmol/mg protein, *P* = 0.037) and WS UGIC patients (0.51 ± 0.40 pmol/mg protein, *P* = 0.042, Figure 3). No correlation was observed between the carnosine levels in RBCs and muscle of WL UGIC patients (Figure S1).

### Effect of skeletal muscle carnosine depletion on the formation of carnosine aldehyde conjugates

**Figure 4:**
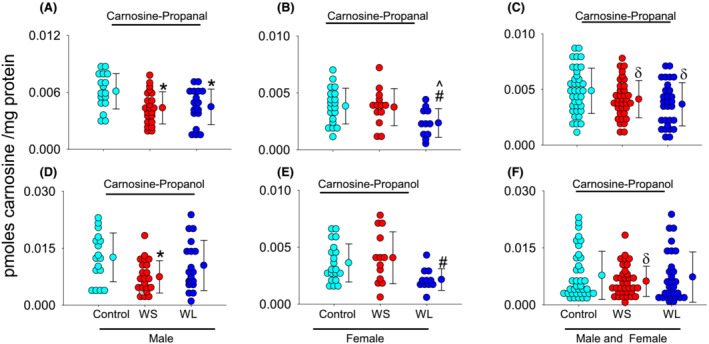
Carnosine aldehyde conjugates detected in the rectus abdominus muscle. Levels of carnosine propanal in the (A) male: Controls (*n* = 17), weight stable (WS; *n* = 22) and weight losing (WS; *n* = 19) upper gastrointestinal cancer (UGIC) patients. (B) Female: Controls (*n* = 20), WS (*n* = 13) and WL (*n* = 11) UGIC patients, and (C) combined: Controls (*n* = 37), WS (*n* = 35) and WL (*n* = 30) UGIC patients. Data are shown as mean ± SD, *P = 0.010 and 0.029 in the male WS and WL UGIC patients versus controls, ^#^ *P* = 0.033 in the female WL UGIC patients versus female controls, and ^^^ *P* = 0.027 versus female WS UGIC patients, and ^δ^ *P* = 0.015 versus all controls. Levels of carnosine propanol conjugates in the (D) male, (E) female, and (F) combined controls, WS and WL UGIC patients. Data are shown as mean ± SD, *P = 0.020 in WS UGIC male patients versus control and ^#^ *P* = 0.008 in the WL UGIC patients versus female controls.

We measured the carnosine aldehyde conjugates, such as carnosine propanal, as well as its reduced product carnosine propanol in the RA muscle. In men, carnosine propanal conjugates were significantly lower in the WS (0.0043 ± 0.001 pmol/mg tissue; *P* = 0.010) and WL UGIC patients (0.0045 ± 0.001 pmol/mg tissue; *P* = 0.029) compared with controls (0.0061 ± 0.001 pmol/mg tissue; Figure 4A). Carnosine propanol conjugates were lower in WS UGIC patients (0.0074 ± 0.004 pmol/mg tissue) compared with controls (0.0125 ± 0.006 pmol/mg tissue; *P* = 0.020) and unchanged in the WL UGIC patients (0.0103 ± 0.006 pmol/mg tissue, Figure 4D). In women, the carnosine propanal conjugates were lower in the WL UGIC (0.0023 ± 0.001 pmol/mg tissue, *P* = 0.033) compared with the WS UGIC patients (0.0037 ± 0.001 pmol/mg tissue) and controls (0.0038 ± 0.001 pmol/mg tissue; *P* = 0.027, Figure 4B). Similarly, carnosine propanol conjugates were significantly lower in the WL UGIC (0.0021 ± 0.0009 pmol/mg tissue) compared with the WS UGIC patients (0.004 ± 0.0022 pmol/mg tissue, *P* < 0.008), but unable to reach statistical significance compared with controls (0.0036 ± 0.0003 pmol/mg tissue, *P* = 0.062 Figure 4E).

To examine which carnosine aldehyde conjugate is predominantly affected by carnosine depletion in both sexes, we combined and analysed both the male and female subjects and found that carnosine propanal conjugates were significantly decreased in the muscle of WL UGIC patients (0.0037 ± 0.001 pmol/mg tissue), compared with controls (0.0048 ± 0.002 pmol/mg tissue; *P* = 0.015, Figure 4C). Carnosine propanol conjugates were unchanged in the WS UGIC patients (0.0061 ± 0.003 pmol/mg tissue) and in the WL UGIC patients (0.0073 ± 0.006 pmol/mg tissue) compared with controls (0.0077 ± 0.006 pmol/mg tissue; Figure 4F).

### Relationship between histidyl dipeptides, carnosine aldehyde conjugates, and muscle wasting

**Figure 5:**
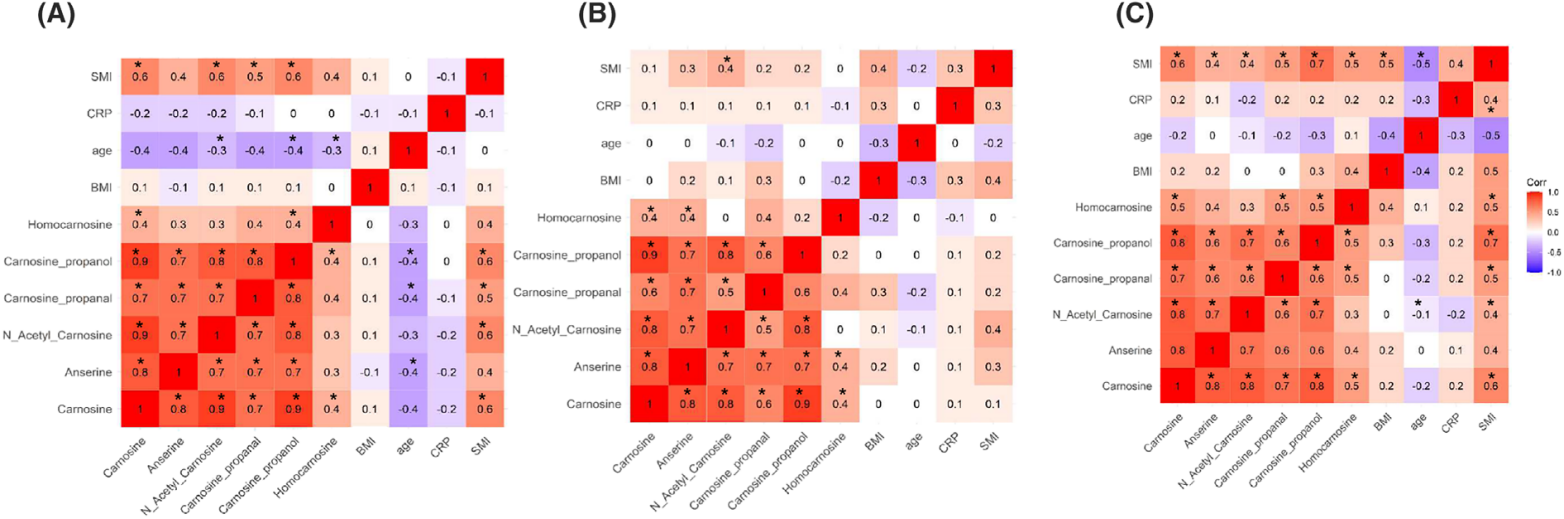
Spearman correlation coefficients between histidyl dipeptides, carnosine aldehyde conjugates and the clinical parameters, skeletal muscle index (SMI), C reactive protein (CRP) and body mass index (BMI) in the male and female subjects. A heat map of (A) male and female controls (*n* = 37), (B) weight stable (WS) male and female upper gastrointestinal cancer (UGIC) patients (*n* = 35), and (C) weight losing (WL) UGIC male and female patients (*n* = 30). Asterisk (*) denotes significant correlation between the carnosine and carnosine aldehyde conjugates with the clinical parameters.

We compared the associations between all histidyl dipeptides and carnosine aldehyde conjugates, with the different clinical characteristics such as age, BMI, CRP, and SMI in controls (*n* = 37). Of all the dipeptides measured, carnosine and N‐acetyl‐carnosine were positively and moderately correlated (Spearman's rho = 0.6), with SMI. Similarly, carnosine aldehyde conjugates, carnosine propanal and carnosine propanol were positively correlated with SMI (Spearman's rho = 0.5–0.6). Negative associations were observed between different histidyl dipeptides and age, whereas no association was observed between carnosine and BMI (Figure 5A). Next, we examined the association between all histidyl dipeptides, and carnosine aldehyde conjugates with the clinical characteristics of WS UGIC patients only (*n* = 35) and found only N‐acetyl carnosine was moderately correlated with SMI (Spearman's rho = 0.4) (Figure 5B). Finally, we examined the association with WL UGIC patients (*n* = 30) and found that carnosine, anserine, and N‐acetyl carnosine were positively and moderately correlated with SMI (Spearman's rho = 0.4–0.6). Carnosine aldehyde conjugates, carnosine propanal and carnosine propanol were also positively correlated with SMI (Spearman's rho = 0.5–0.7) (Figure 5C).

### Effect of upper gastrointestinal cancer on histidyl dipeptide homeostasis

**Figure 6:**
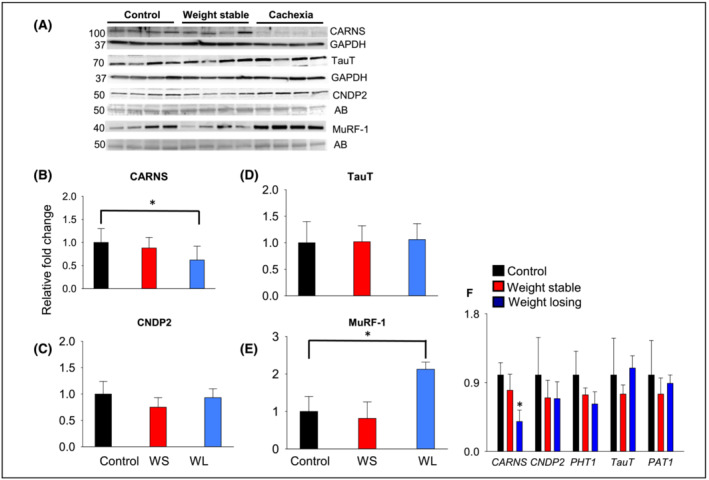
Expression of carnosine transporters, synthesizing and hydrolysing enzymes in the rectus abdominus (RA) muscle. Representative blots for (A) carnosine synthase (CARNS), human synthetic taurine transporter (TauT), carnosinase (CNDP2) and muscle RING finger protein (MuRF‐1) in the RA muscle of controls, weight stable (WS) and weight losing (WL) upper gastrointestinal cancer patients. Bands are normalized to GAPDH or amido black (AB) (B–E). (F) Relative mRNA expression of *CARNS*, *CNDP2*, peptide/histidine transporter (*PHT1*), *TauT* and amino acid transporter (*PAT1*) in the RA muscle. *P < 0.04 versus all controls. Data are presented as mean ± SD, *n* = 10 samples in each group.

To determine how carnosine may be depleted in cachectic muscle, we examined the contribution of synthesis (CARNS),^21^ transport (TauT),^22^ and hydrolysis by CNDP2.^23^ We found that CARNS expression was significantly decreased, whereas TauT and CNDP2 expression remained unchanged in WL UGIC muscle compared with the controls and WS UGIC patients (Figure 6A–D). We measured the expression of muscle specific atrophic marker, muscle ring finger‐1 (MuRF‐1), which was increased in WL UGIC muscle compared with controls and WS UGIC patients (Figure 6E), thus further supporting the patient group allocation. As protein degradation is a central feature of cancer cachexia, we next compared the mRNA levels of *CARNS*, *TauT*, *PHT1*, and *CNDP2* between the three groups. Similar to changes observed with protein expression, *CARNS* mRNA expression was significantly lower in the muscle of WL UGIC patients, compared with the controls and WS UGIC patients (Figure 6F).

### Effect of endogenous carnosine production on atrophic signalling pathways in skeletal muscle myotubes

**Figure 7:**
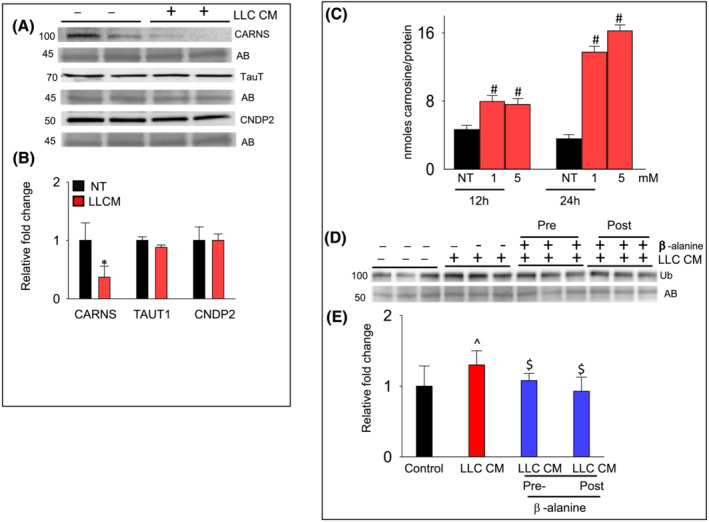
β‐Alanine and Lewis lung carcinoma cells (LLC) conditioned media (CM) treatment of differentiated skeletal muscle myoblasts (myotubes). Myotubes were treated with LLC CM for 72 h. (A) Cell lysate was analysed for (B) CARNS, TauT, and CNDP2 expression. Data are mean ± SD, *n* = 5 samples in each group, *P < 0.02 versus non‐treated cells. (C) Levels of carnosine detected by LC–MS/MS in the β‐alanine treated (1 and 5 mM; 12 and 24 h treatment), C2C12 cells. Data are presented as mean ± SD, ^#^ *P* < 0.04 versus non‐treated cells (NT). (D) Levels of the ubiquitinated proteins in myotubes either pretreated with β‐alanine (1 mM) for 12 h followed by LLC CM treatment for 72 h, or post β‐alanine (1 mM) and LLC CM treatments. (E) Data are presented as mean ± SD, *n* = 5 samples in each group, ^*P* < 0.05 versus controls, ^$^ *P* < 0.05 versus LLC CM treated cells.

We treated differentiated C2C12 cells (myotubes) with LLC CM that mimic the typical conditions of cachexia. Treatment of myotubes with LLC CM for 72 h decreased CARNS expression, whereas the expression of TauT and CNDP2 remained unchanged when compared with the controls (Figure 7A,B). Thus, in accordance with the observations in human skeletal muscle, *in vitro* atrophying stimuli also affected carnosine synthesis.

Finally, to explore whether increasing endogenous carnosine production could increase resistance to LLC CM‐induced atrophy, we treated myotubes with β‐alanine (1–5 mM) for 12 to 24 h and measured levels of intracellular carnosine. Carnosine levels in the β‐alanine myotubes were increased 2‐ to 3‐fold compared with non‐treated myotubes (Figure 7C). Next, we pretreated myotubes with β‐alanine followed by LLC CM treatment for 72 h or with β‐alanine and LLC CM treatment for 72 h and analysed for protein ubiquitination, a marker for protein degradation. Significantly, both the pre‐ and immediate ‐β‐alanine treatments in the LLC CM treated myotubes decreased protein ubiquitination, compared with LLC CM treated myotubes only (Figure 7D,E).

## Discussion

Cancer cachexia is a severe and debilitating pathology characterized by diminished response to anticancer drugs, poor quality of life and increased mortality.^1^, ^2^ However, there are no biomarker‐based approved therapies, which could be targeted to prevent the progression of this syndrome. Given that protein degradation pathways are sensitive to [pH]_i_ and oxidative stress, we examined the role of histidyl dipeptides, which are abundant in skeletal muscle and possess unique chemical properties that regulate both [pH]_i_ and oxidative stress. We found that carnosine, the predominant dipeptide in human skeletal muscle demonstrated differential responses; carnosine levels were decreased in the WS and WL upper gastrointestinal cancer (UGIC) male patients, whereas in women, its levels were decreased in the WL UGIC patients only. Similarly, other histidyl dipeptides also showed differential responses; such as homocarnosine was decreased in the male WS UGIC patients, anserine and N‐acetylcarnosine remained unchanged, whereas in women; anserine and N‐acetyl carnosine were decreased in the WL UGIC patients. Further, we found that among all the histidyl dipeptides, carnosine was the only dipeptide decreased in the muscle of WL UGIC patients of both sexes.

Our results show that carnosine levels were much lower in RBCs collected from the cachectic patients than the controls and WS UGIC population. However, no associations were observed between carnosine levels in the RBCs and skeletal muscle. Considering that RBCs occupy about half of blood volume, exhibit active metabolism with a lifespan of ~4 months and carnosine levels are altered by diet intake,^18^, ^24^ it would be interesting to measure carnosine in RBCs prospectively at different stages of cancer, measure the intake of carnosine from diet, and then determine whether carnosine in RBCs could identify patients susceptible to developing cachexia.

We also found that carnosine aldehyde conjugates were decreased in WL UGIC patients, suggesting that carnosine depletion due to cancer could decrease the ability of skeletal muscle to remove reactive products of lipid peroxidation, which may perpetuate muscle wasting and protein degradation. In WL UGIC patients and C2C12 cells treated with tumour‐derived cytokines, CARNS expression was decreased, suggesting that the synthesis of these dipeptides, in particular, could be affected by cancer‐derived cytokines. The functional significance of carnosine depletion is underscored by our observation that carnosine levels in the muscle were positively associated with the SMI, in cachectic patients. Although such association does not imply causation, our observation that enhancing endogenous carnosine production in C2C12 cells by β‐alanine supplementation attenuated cachectic signalling suggests that carnosine depletion may be a significant factor contributing to protein degradation and muscle wasting. Taken together, these results suggest that cancer‐derived cytokines deplete skeletal muscle carnosine by downregulating CARNS and that carnosine depletion compromises the ability of muscle to remove endogenous oxidants, and plays a significant role in stimulating protein degradation and muscle wasting. Importantly, these findings suggest that alleviating or preventing carnosine depletion in the skeletal muscle of cancer patients could be an effective intervention to improve cancer‐induced cachexia.

The severity of cachexia varies from one individual to another. Even with the existence of similar tumours, growth patterns and identical origins, there remains heterogeneity in the clinical presentation of cachexia. Numerous factors contribute to the variable prevalence of cancer cachexia including, sex, age, genetic factors, and comorbidities. While men generally have higher skeletal muscle mass and higher carnosine content than women, male cancer patients have greater weight and muscle loss compared with women.^25^ We found that in male WS UGIC patients, carnosine levels were lower than those in controls, whereas these levels remained unchanged in female WS UGIC patients. A possible explanation for the observed sex differences in carnosine depletion could be the low circulating testosterone levels, observed in men with metastatic malignancy.^26^ Testosterone regulates carnosine synthesis in muscle^27^ whereas hypogonadism decreases carnosine in male individuals.^28^ Therefore, a potential interplay between hypogonadism and carnosine regulation by testosterone, might enhance the susceptibility of male cancer patients to lose carnosine in muscle and therefore develop more pronounced cachexia.

Hypercatabolism of muscle proteins is one of the main characteristics of muscle cachexia. The accelerated loss of muscle protein has been attributed to the activation of several intracellular proteolytic pathways, such as ubiquitin/proteasome, autophagy and caspases.^29^ Many lines of evidence have implicated that ROS are critical activators of protein degradation pathways. Numerous studies with human cancer patients and animal models of cancer cachexia show that during carcinogenesis, ROS generation and the formation of oxidized proteins are increased, activities of antioxidant enzymes are reduced, and the expression of NADPH oxidase 4, a key regulator of ROS production, is increased in cachectic muscle.^5^, ^6^, ^7^ The hypothesis that increased ROS production contributes to the pathological changes in cachexia is further supported by the observation that mice lacking antioxidant enzyme superoxide dismutase display oxidative damage, and experience accelerated age‐associated muscle atrophy.^30^ To counteract oxidative stress, muscle synthesizes nucleophiles, such as glutathione and histidyl dipeptides, which quench ROS and remove reactive products generated by ROS, particularly lipid peroxidation products. However, carnosine levels in the muscle are much higher than those of glutathione and it can additionally buffer intracellular protons^31^, ^32^ and thereby prevent both the ROS generation and buffer [pH]_i_, under hypoxic conditions. The homeostasis of carnosine in healthy muscle is tightly regulated with a biological variation of approximately 5% over a period of 15 weeks^33^; however, its levels are significantly depleted in conditions associated with muscle wasting, such as multiple sclerosis and severe chronic obstructive pulmonary disease.^34^, ^35^ However, a direct association of carnosine with muscle wasting has not been reported previously. Therefore, our observations showing that carnosine was reduced in the muscle of cachectic patients and carnosine levels were positively associated with SMI are consistent with the notion that carnosine depletion plays a causative role in cachexia.

Previous studies with humans and in animal models of chronic kidney disease have found that metabolic acidosis causes loss of protein stores and muscle wasting^36^ and that correction of acidosis improves protein catabolism in muscle.^37^ In this regard, histidyl dipeptides, especially carnosine, which play a major role in buffering [pH]_i_, during exercise and under ischemic conditions,^16^ therefore could diminish protein catabolism by buffering [pH]_i_. We found that MURF1 expression, a marker of ubiquitin proteasome pathway, was increased in the WL UGIC patients, which may be a response to the altered buffering potential or [pH]_I_ in cachectic muscle due to carnosine depletion. Thus, carnosine loss in muscle during cancer could have critical consequences on the antioxidant status and buffering potential, which could subsequently exacerbate protein catabolism, and alter the muscle metabolism of cancer patients.

In cachectic patients, carnosine could be depleted *via* several mechanisms. For instance, decrease in the expression of transporters would diminish the transport of amino acids and carnosine synthesis.^22^ However, our examination of cachectic muscle and treatment of myotubes with LLC CM showed no effect on amino acid transporters such as Taut and PHT1. Likewise, expression of CNDP2, which hydrolyses carnosine,^23^ remained unaffected in the muscle of WL UGIC patients and LLC CM treated myotubes, indicating that transport and hydrolysis are unlikely to contribute to carnosine depletion. Instead, the expression of CARNS, which synthesizes carnosine,^21^ was decreased in cachectic muscle and LLC CM treated myoblasts, suggesting that synthesis of these dipeptides is particularly targeted during muscle wasting. Nevertheless, even though our data show that CARNS expression is lower in WL UGIC patients, and are reduced by factors secreted by lung cancer, additional studies are needed to explore the influence of other cancer types.

Extensive evidence shows that β‐alanine supplementation enhances carnosine in tissues, such as muscle and heart.^17^, ^32^ We have found that treatment of myotubes treated with β‐alanine, increased endogenous carnosine levels 2‐ to 3‐fold. Importantly, both the pre and post treatment of β‐alanine prevented the induction of protein ubiquitination in LLC CM treated myotubes. Although the mechanisms by which carnosine alleviates atrophic signalling needs to be elucidated, we speculate that the antioxidant and buffering abilities of this multifunctional dipeptide prevents the onset of protein degradation pathways.

### Study limitations

The present study is the first to show that histidyl dipeptides are affected by cancer‐induced muscle wasting and a differential response of these dipeptides to sexual dimorphism. However, there are limitations to the study. For instance, it has been reported that carnosine levels in the aged individuals (64.58 ± 5.84 years) are ~50% (3.75 ± 1.56 nmol) lower than in young individuals (age 29.4 ± 6.72 years; carnosine 6.43 ± 1.76 nmol).^38^ In our study, the WS UGIC patients were 63 ± 9 years and controls were 53 ± 12 years old, which is within the age range of old subjects that were compared with young subjects for the carnosine levels.^38^ Although the controls in our study were younger compared with the WS UGIC patients (*P* < 0.05, Table 1), we did not observe any significant differences in carnosine levels between the two groups (Figures 2 and 3), suggesting that age might act as a confounder only when comparisons are made with very young individuals. Hence, a study with age matched controls would be beneficial to examine whether the decrease in carnosine levels observed in the WL UGIC patients is independent of age.

Previous reports show that carnosine in RBCs is a biomarker of aging and frailty.^39^, ^40^ Our results show carnosine levels are decreased in the RBCs of WS UGIC patients; however, no association was observed with the muscle carnosine. Given that carnosine levels are altered by diet,^18^, ^24^ and as yet, no direct link shows that depletion of histidyl dipeptides in muscle is the underlying cause of carnosine depletion in RBCs, it would be interesting to perform a prospective study, measure dietary intake of carnosine also in cancer patients, and then identify patients susceptible to developing cachexia, on the basis of carnosine levels in RBCs.

## Conclusion

Protein degradation pathways, such as autophagy and ubiquitin proteasome, activated by oxidative stress and decrease in [pH]_I_, respectively, are key signalling mechanisms involved in protein catabolism. Carnosine, a small multifunctional nucleophilic compound, quenches ROS, removes toxic aldehydes and buffers [pH]_I_. Our observations showing carnosine is depleted in human cancer cachectic muscle suggests that such depletion could alter antioxidant defences and [pH]_i_ buffering capacity of muscle in cancer patients, and could potentially contribute to the patho‐biochemistry of muscle atrophy. Typically, the clinical manifestations of cachexia, and the ability to treat them, are complicated by the necessities and side‐effects of tumouricidal therapy. Numerous *in vitro* and *in vivo* studies suggest that carnosine inhibits proliferation of different cancers and notably carnosine inhibits growth of human gastric cells,^41^, ^42^ indicating a potential dual role of carnosine in cancer therapy. Evidence that carnosine in skeletal muscle can be elevated by feeding and exercise, that it exhibits anti‐neoplastic properties, and that increasing carnosine in the skeletal muscle myoblast attenuates cachectic signalling, suggests that carnosine could be used as an intervention to combat the paraneoplastic effects of cancer and the onset of cachexia in both sexes. Our finding suggests that carnosine in RBCs could serve as a readily accessible biomarker of cancer cachexia, which could inform the design of future clinical trials as well as the development of new therapeutic interventions.

## Conflict of interest statement

None declared.

## Supporting information


**Data S1.** Supporting Information

**Fig. S1.** Relation of carnosine concentration in the RBCs and muscle of weight losing upper gastrointestinal cancer patents.
