# Supplementary material for: Skeletal muscle analysis of cancer patients reveals a potential role for carnosine in muscle wasting
Source: J Cachexia Sarcopenia Muscle. 2023 May 18;14(4):1802–14. doi: 10.1002/jcsm.13258 (PMC10401540; doi:10.1002/jcsm.13258)
Supplement: Supplementary file 1 — Data S1. Supporting Information [file JCSM-14-1802-s002.docx]

**Supplemental Material and Methods**

**Body composition analysis**

Cachexia was defined according to consensus definition: specifically, >2% weight loss combined with low muscularity over a period of 6 months, which has been shown in our previous studies to demonstrate histological muscle wasting [1, 2]. Body weight was measured with patients lightly clothed using a beam scale (Seca, UK). Height was measured using a standard wall mounted measure. Weight change was calculated as the percentage difference between stable pre-morbid weight (self-reported by the patient) and measured weight on assessment. BMI was calculated using the formula:

BMI (kg/m^2^) = weight (kg)/height (m)^2^

**Muscle biopsy**

Rectus abdominis (RA) muscle is a well characterized tissue used for the study of cancer cachexia in humans, which shows evident changes of wasting, such as atrophy [3].  Approximately 1cm^2^ biopsies of RA were taken using sharp dissection, without the use of electrocautery. Samples were cleaned of fat/blood/fibrous tissue, placed in a tube (NUNC Cryotubes, Sigma-Aldrich Co. LLC) snap frozen, and stored at −80°C before analysis.

**Histidyl dipeptide profiling of RA muscle and RBCs**

Histidyl dipeptides were analyzed by LC/MS as described previously [4]. Muscle homogenate (5%) was prepared in extraction solution HCl (10 mM) using internal standards (IS), L-carnosine-d_4_, tyrosyl-histidine and L-anserine-d_4_. Following homogenization, samples were sonicated on ice, centrifuged at 4°C (10 min, 16,000×g). Before analysis the collected supernatants were diluted 50 × in a 75:25 acetonitrile:water mixture and injected into a Waters ACQUITY UPLCH-Class Systems with a Xevo TQ-S microtriple quadrupole. Chromatograms were acquired using the transitions: carnosine 227→110 m/z, anserine 241→109 m/z, homocarnosine 241→156 m/z, N-acetylcarnosine 268→156 m/z, carnosine propanal 283→166 m/z, and carnosine propanol 285→110 m/z, carnosine-d_4_ 231→110 m/z, anserine d_4_ 245 →110 m/z in multiple reaction monitoring (MRM) mode. For RBCs, ~40-50 µl of packed RBC were mixed with extraction buffer (375 µl, 55% methanol and IS) spun in centrifugal filter units (3000MW, Amicon Ultra) at 16000 × g and analyzed on LC-MS/MS as mentioned above.

**Immunoblotting**

Immunoblots were developed using anti-CARNS1 (1:1000; COSMOBIO), anti-CNDP2 (1:1000; Abclonal), anti-TAUT (1:1000; Abclonal), anti-ubiquitin (1:1000; Sigma Life Sciences), and anti-MURF1 (1:1000; Abclonal) antibodies. Band intensity was quantified by using Imagequant TL software and normalized to GAPDH and Amido-black staining.

**Quantitative Polymerase Chain reaction**

Expression of the genes encoding *CARNS1, CNDP2, PHT1, PAT1 and TAUT* were determined using quantitative RT-PCR and primers described previously [5]. Results were normalized to HPRT1 and expressed according to the comparative C_t_ method, where the C_t_ values of gene of interest were compared to the controls. Measurements were made using Prism 7900 HT (Applied Biosystem).

**Treatment of the murine myotubes with Lewis Lung carcinoma conditioned medium (LLC CM)**

To determine whether tumor cytokines directly affect the expression of proteins and transporters involved in histidyl dipeptide homeostasis, fully differentiated C2C12 cells (myotubes) were treated with LLC CM. Normal culture medium was used as control. For β-alanine pre and post treatment, 1-5 mM of β-alanine dissolved in the medium was added 12 h prior and immediately before the LLC CM treatment. Intracellular carnosine formation in the myotubes was measured by LC-MS/MS as described above. Briefly, following the β-alanine treatment, the myotubes were washed with cold PBS and homogenized (30 s, 3m/s, Bead Ruptor Elite, Omni International), in the extraction buffer containing HCl (10 mM) and internal standards, carnosine-d_4_ and L-anserine-d_4_. Following homogenization, the samples were centrifuged at 4°C (10 min, 16,000 × g), and the supernatants were analyzed on LC-MS/MS. To examine whether the expression of transporters, CARNS and ubiquitination of proteins is affected by LLC-CM treated, we performed Western blot and developed the immunoblots with the antibodies CARNS, CNDP2, TAUT and ubiquitin as described above and in supplemental information.

**Supplemental Legend**

**Table I.** Proportions of sex, type of cancer, tumor stage and nodal classification.

**Fig.1**. Relation of carnosine concentration in the RBCs and muscle of weight losing upper gastrointestinal cancer patents**.**

**References**

1. Boehm I, Miller J, Wishart TM, Wigmore SJ, Skipworth RJ, Jones RA, et al. Neuromuscular junctions are stable in patients with cancer cachexia. J Clin Invest. 2020;130:1461-5.

2. Johns N, Hatakeyama S, Stephens NA, Degen M, Degen S, Frieauff W, et al. Clinical classification of cancer cachexia: phenotypic correlates in human skeletal muscle. PLoS One. 2014;9:e83618.

3. Anoveros-Barrera A, Bhullar AS, Stretch C, Esfandiari N, Dunichand-Hoedl AR, Martins KJB, et al. Clinical and biological characterization of skeletal muscle tissue biopsies of surgical cancer patients. J Cachexia Sarcopenia Muscle. 2019;10:1356-77. doi:10.1002/jcsm.12466

4. Hoetker D, Chung W, Zhang D, Zhao J, Schmidtke VK, Riggs DW, et al. Exercise alters and beta-alanine combined with exercise augments histidyl dipeptide levels and scavenges lipid peroxidation products in human skeletal muscle. J Appl Physiol (1985). 2018.

5. Boakye AA, Zhang D, Guo L, Zheng Y, Hoetker D, Zhao J, et al. Carnosine Supplementation Enhances Post Ischemic Hind Limb Revascularization. Front Physiol. 2019.
