# Supplementary figures and images for: Skeletal muscle analysis of cancer patients reveals a potential role for carnosine in muscle wasting
Source: J Cachexia Sarcopenia Muscle. 2023 May 18;14(4):1802–14. doi: 10.1002/jcsm.13258 (PMC10401540; doi:10.1002/jcsm.13258)

## Slide 1
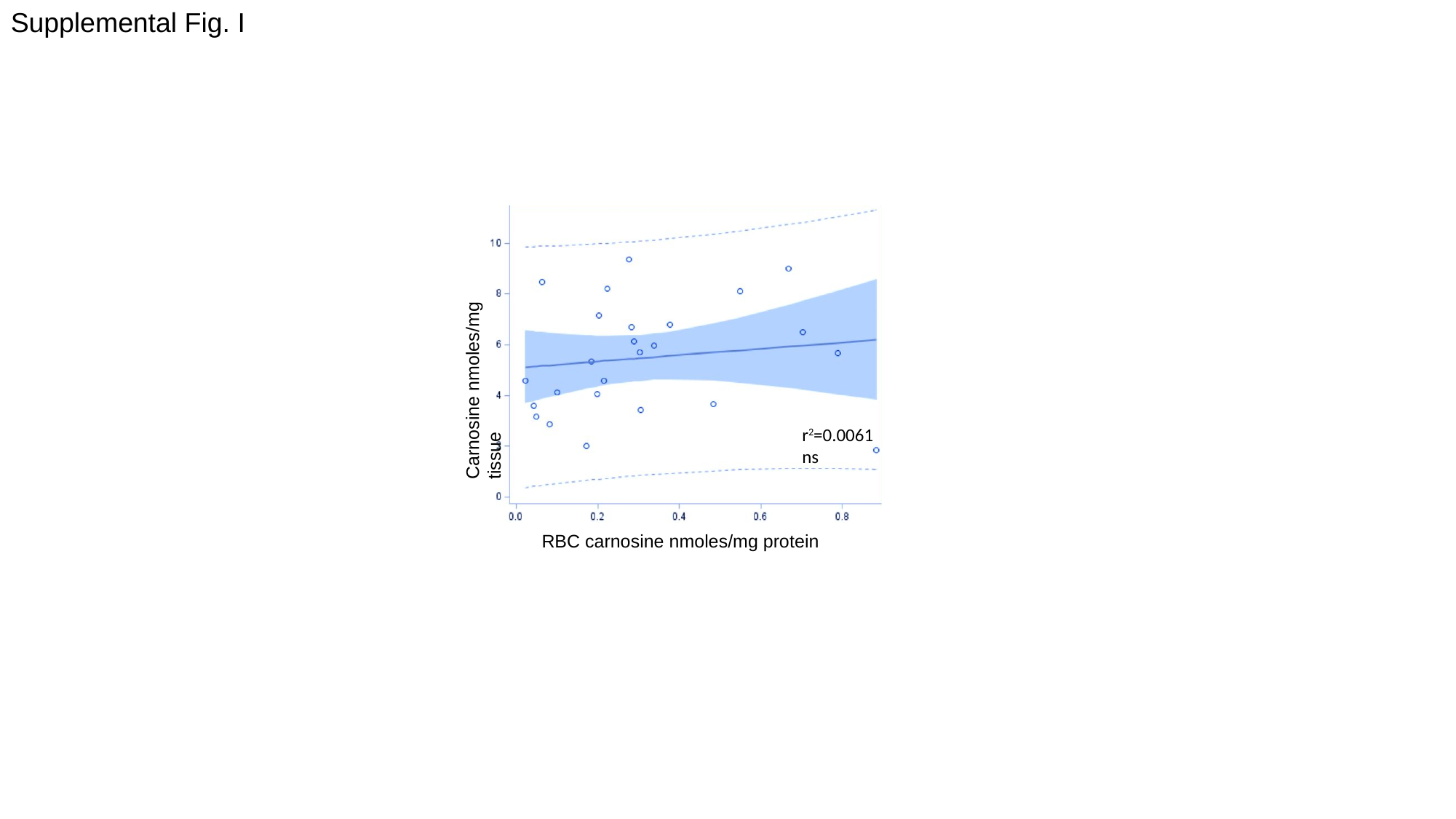

Supplemental Fig. I
Carnosine nmoles/mg tissue
r2=0.0061
ns
RBC carnosine nmoles/mg protein

Supplement: Supplementary file 2 — Fig. S1. Relation of carnosine concentration in the RBCs and muscle of weight losing upper gastrointestinal cancer patents. [file JCSM-14-1802-s001.pptx]
